# Neuropsychiatric Manifestations of Lyme Disease: A Literature Review of Psychiatric and Cognitive Impacts

**DOI:** 10.15388/Amed.2025.32.1.17

**Published:** 2025-02-18

**Authors:** Gabija Šegždaitė, Odeta Aliukonytė, Kamilė Pociūtė

**Affiliations:** 1 Medical Academy, Faculty of Medicine, Lithuanian University of Health Sciences, Kaunas, Lithuania; 2 Faculty of Medicine, Vilnius University, Vilnius, Lithuania; 3 Clinic of Psychiatry, Institute of Clinical Medicine, Faculty of Medicine, Vilnius University, Vilnius, Lithuania

**Keywords:** Lyme disease, borreliosis, psychiatric disorders, neuropsychiatric symptoms, Laimo liga, boreliozė, psichikos sutrikimai, neuropsichiatriniai simptomai

## Abstract

**Background:**

Lyme disease can lead to neuropsychiatric symptoms like depression, anxiety, and cognitive issues, often mimicking primary psychiatric disorders. This paper examines the connection between Lyme disease and neuropsychiatric outcomes to improve diagnosis and treatment.

**Materials and Methods:**

The *PubMed* database was searched for scientific literature sources. Publications published in English in 2019–2024 were selected. All psychiatric symptoms and disorders found to be associated with Lyme disease were included. For neurological symptoms, the analysis focused on studies addressing cognitive dysfunction.

**Results:**

Acute neuroborreliosis may have minimal effects on the cognitive function and typically resolves well with treatment. However, some studies suggest that Lyme disease can affect the patients’ cognitive abilities, leading to impairments in verbal fluency, attention, and memory, with a few isolated dementia-like cases highlighting the need for careful diagnosis. Nevertheless, recent large-scale studies show no increased risk of dementia. Regarding psychiatric symptoms, findings are also inconsistent, with some studies suggesting an increased risk of depression, anxiety, sleep disturbances, and other mental health conditions, while others find no such association.

**Conclusions:**

There is no strong evidence supporting Lyme disease’s role in long-term cognitive or psychiatric disorders. However, an early diagnosis and timely antibiotic treatment remain crucial in minimizing long-term consequences and improving patient outcomes.

## Abstract (it)

**Įvadas:**

Laimo liga gali sukelti neuropsichiatrinių simptomų, kaip antai depresiją, nerimą ir kog-nityvinius sutrikimus, dažnai imituojančius pirminius psichikos sutrikimus. Šiame straipsnyje nagrinėjamas Laimo ligos ir neuropsichiatrinių padarinių ryšys, siekiant pagerinti ligos diagnostiką ir gydymą.

**Medžiaga ir metodai:**

*PubMed* duomenų bazėje ieškota mokslinės literatūros šaltinių. Buvo atrinktos 2019–2024 metais anglų kalba paskelbtos publikacijos. Įtraukti visi psichiatriniai simptomai ir sutrikimai, kurie, kaip nustatyta, yra susiję su Laimo liga. Kalbant apie neurologinius simptomus, analizuoti tyrimai, kuriuose nagrinėti kognityvinės funkcijos sutrikimai.

**Rezultatai:**

ūminė neuroboreliozė gali turėti minimalų poveikį kognityvinėms funkcijoms ir paprastai tinkamai gydant praeina. Tačiau kai kurie tyrimai rodo, kad Laimo liga gali turėti įtakos pacientų pažintiniams gebėjimams, dėl to gali sutrikti žodžių sklandumas, dėmesys ir atmintis, o keli pavieniai į demensiją panašūs atvejai rodo, kad reikia kruopščiai diagnozuoti. Vis dėlto naujausi didelės apimties tyrimai nerodo padidėjusios demensijos rizikos. Psichiatrinių simptomų tyrimo rezultatai taip pat nėra nuoseklūs: vienų tyrimų duomenimis, yra didesnė depresijos, nerimo, miego sutrikimų ir kitų psichikos sveikatos sutrikimų rizika, o pagal kitus tyrimus tokio ryšio nerasta.

**Išvados:**

Nėra tvirtų įrodymų, patvirtinančių Laimo ligos vaidmenį ilgalaikiams pažinimo ar psichikos sutrikimams atsirasti. Tačiau ankstyva ligos diagnozė ir pacientų gydymas laiku antibiotikais tebėra labai svarbūs siekiant sumažinti ilgalaikius padarinius ir pagerinti pacientų gydymo rezultatus.

## Introduction

*Lyme Borreliosis* (LB) is a tick-borne infectious disease caused by the spirochete bacteria *Borrelia burgdorferi sensu lato* [1]. LB is the most common tick-borne disease in the United States and Europe, but the disease burden is incompletely described [1, 2]. Recent estimates suggest that approximately 476,000 people may be diagnosed and treated for Lyme disease in the United States each year, likely because this number includes patients who are treated based on clinical suspicion but do not actually have Lyme disease [2]. One systematic review showed that the highest national incidence of LB in Europe was in Estonia, Lithuania, Slovenia, and Switzerland (>100 cases/100,000 population), followed by France and Poland (40–80 cases/100,000 population), Finland and Latvia (20–40 cases/100,000 population) [3]. In Europe, the most common and significant tick is *Ixodes Ricinus* in terms of its wide ecosystem distribution and the diversity of transmitted pathogens [4]. Over the past few decades, the incidence of tick-borne diseases in Europe has grown, driven by biotic and abiotic factors [5]. The evidence shows that global warming is one of the most important factors behind the redistribution of tick populations and the outbreak of tick-borne diseases [6, 7].

The disease is usually recognized by its distinctive early symptoms, such as *erythema migrans*, a circular, spreading rash at the site of the tick bite, and flu-like symptoms, such as fever, fatigue, and muscle aches [8]. Nevertheless, LD can occur in different body systems, especially when untreated or inadequately treated. The infection can spread to the joints and cause arthritis [9], in the heart and cause carditis [10], in the nervous system and cause neuroborreliosis [11]. There are three clinically determined stages of LD. The first is the early localized stage, usually manifested by a rash of *erythema migrans*. Stage 2 is defined as an early stage of development with musculoskeletal, cardiovascular, and neurological symptoms. Stage 3, the late dissemination stage, is associated with increased neurological signs. The late stage occurs when LD is untreated, and the bacteria have spread to the *Central Nervous System* (CNS), resulting in significant neuropsychiatric symptoms and cognitive decline [12]. Studies show that neuropsychiatric and cognitive symptoms tend to be chronic, even after recovery [13], and may include mental symptoms such as impairments in working memory, slower processing of information, also known as ‘brain fog’, impairments in verbal learning/memory, non-verbal learning/memory, vigilance, visual-constructive and frontal executive function [14]. Suicidal ideation, homicidal tendencies, aggression, depressive symptoms, and anxiety may also be present [14].

Consequently, Lyme disease can mimic psychiatric illnesses and cause emotional and cognitive disturbances, and early diagnosis and intervention are key factors in avoiding long-term complications and improving patient outcomes. This article synthesizes existing research on the neuropsychiatric effects of Lyme disease, aiming to enhance clinical understanding and improve the patient care. The review explores psychiatric symptoms linked to Lyme disease, the impact of early-onset Lyme disease on the cognitive function, cognitive deficits in *Post-Treatment Lyme Disease Syndrome* (PTLDS), associations with dementia, and rare neuropsychiatric manifestations.

## Methods

The *PubMed* database was searched for scientific literature sources. Publications published in English from 2019 to 2024 were selected by using the following keyword combination: (Lyme disease OR Borrelia infection OR tick-borne OR neuroborreliosis) AND (mental disorders OR psychiatric symptoms OR psychiatry OR mental health OR depression OR anxiety OR psychosis OR neuropsychiatric OR cognitive OR dementia OR neurology OR neurological symptoms). All Lyme-related mental symptoms and disorders were reviewed. For neurological symptoms, the focus was on studies related to cognitive impairment. A total of 883 articles were found. Excluding reviews, non-English articles, off-topic, and duplicates, 26 publications remain.

Based on the data obtained, we have divided the results into 5 sub-topics: (1) The relationship between early-onset Lyme disease and cognitive impairment; (2) Cognitive impairment in post-treatment Lyme disease syndrome patients; (3) Lyme disease in relation to the development of dementia; (4) Psychiatric symptoms in patients with Lyme disease; and (5) Rare neuropsychiatric presentations of Lyme disease. Summarized publication data are shown in Table 1.

**Table 1:** Summary characteristics of the articles

Author, date	Study	Chapter in which the article is included	Type of the study	Sample of subjects with Lyme disease	Findings regarding neuropsychiatric symptoms
Gorlyn, M. et al., 2023	Language Fluency Deficits in Post-treatment Lyme Disease Syndrome	Cognitive impairment in post-treatment Lyme disease syndrome (PTLDS) patients	Cross-sectional study	n=131	Lower verbal fluency in comparison with healthy controls
Marvel, C.L. et al., 2022	A multimodal neuroimaging study of brain abnormalities and clinical correlates in post treatment Lyme disease	Cognitive impairment in post-treatment Lyme disease syndrome (PTLDS) patients	Cross-sectional study	n=30	Slower on the working memory task but accuracy remained the same as in controls
Geebelen, L. et al., 2022	Non-specific symptoms and post-treatment Lyme disease syndrome in patients with Lyme borreliosis: a prospective cohort study in Belgium (2016–2020)	Cognitive impairment in post-treatment Lyme disease syndrome (PTLDS) patients	Cohort study	n=1358	Memory problems, difficulty concentrating, and trouble finding words did not significantly differ from controls
Andreassen, S. et al., 2022	Cognitive function in patients with neuroborreliosis: A prospective cohort study from the acute phase to 12 months post treatment	Cognitive impairment in post-treatment Lyme disease syndrome (PTLDS) patients	Cohort study	n=58	Neuroborreliosis patients showed no decline in attention or processing speed 12 months post-treatment
Fallon, B. A. et al., 2021	Lyme Borreliosis and Associations With Mental Disorders and Suicidal Behavior: A Nationwide Danish Cohort Study	Psychiatric symptoms in patients with Lyme disease	Cohort study	n=12156	Increased risk of mental disorders, affective disorders, suicide attempts, and suicide compared with those without Lyme borreliosis
Tetens, M.M. et al., 2021	No associations between neuroborreliosis in children and psychiatric neurodevelopmental disorders: a nationwide, population-based, matched cohort study	Psychiatric symptoms in patients with Lyme disease	Cohort study	n=1132	No associations found between neuroborreliosis and attention deficit disorder, learning disabilities, or intellectual developmental disorders in children
Vargas, S.E. et al., 2021	Characterizing the Symptoms of Patients with Persistent Post-Treatment Lyme Symptoms: A Survey of Patients at a Lyme Disease Clinic in Rhode Island	Psychiatric symptoms in patients with Lyme disease	Cross-sectional study	n=52	People with persistent post-Lyme treatment symptoms experienced mild depression, anxiety, and sleep disturbances. Reported symptoms exceeded those in some clinical populations, including cancer and chronic pain patients
Voitey, M. et al., 2020	Functional signs in patients consulting for presumed Lyme borreliosis	Psychiatric symptoms in patients with Lyme disease	Case-control study	n=48	Lyme borreliosis patients reported memory issues, irritability, and sadness less frequently compared to control
Maxwell, S.P., 2020	The Elusive Understanding of Lyme Disease in Non-Endemic Geographic Areas: An Exploratory Survey of Patients With Chronic Symptoms in Texas	Psychiatric symptoms in patients with Lyme disease	Cross-sectional study	n=95	Anxiety and depression observed in patients who self-report a diagnosis with Lyme disease
Tetens, M.M. et al., 2021	Assessment of the Risk of Psychiatric Disorders, Use of Psychiatric Hospitals, and Receipt of Psychiatric Medication Among Patients With Lyme Neuroborreliosis in Denmark	Psychiatric symptoms in patients with Lyme disease	Cohort study	n=2897	Patients with Lyme neuroborreliosis did not have an increased risk of developing psychiatric diseases
Rožič, M. et al., 2019	Lyme Neuroborreliosis in Children: Etiology and Comparison of Clinical Findings of Lyme Neuroborreliosis Caused by Borrelia garinii and Borrelia afzelii	Psychiatric symptoms in patients with Lyme disease	Cohort study	n=153	Lyme neuroborreliosis, *B. garinii* causes more pronounced CNS inflammation than ***B. afzelii*** but did not lead to more frequent neuropsychiatric symptoms
Hündersen, F. et al., 2021	Neuropsychiatric and Psychological Symptoms in Patients with Lyme Disease: A Study of 252 Patients	Psychiatric symptoms in patients with Lyme disease The relationship between early-onset Lyme disease and cognitive impairment	Cross-sectional study	n=252	Compared to healthy controls, Lyme disease patients reported lower quality of life, poorer sleep, cognitive impairments, particularly in attention and memory, and more depressive symptoms
Bransfield, R.C. et al., 2020	A Clinical Diagnostic System for Late-Stage Neuropsychiatric Lyme Borreliosis Based upon an Analysis of 100 Patients	Psychiatric symptoms in patients with Lyme disease The relationship between early-onset Lyme disease and cognitive impairment	Cohort study	n=100	Impairments in attention span, memory, processing, behavior, executive, emotional, psychiatric, and neurological functioning compared to pre-infection data
Maxwell, S.P. et al., 2022	Neurological Pain, Psychological Symptoms, and Diagnostic Struggles among Patients with Tick-Borne Diseases	Psychiatric symptoms in patients with Lyme disease The relationship between early-onset Lyme disease and cognitive impairment	Cross-sectional study	n=43	Anxiety, depression, panic attacks, hallucinations, and delusions are commonly reported among patients diagnosed with a tick-borne disease
Berende, A. et al., 2019	Cognitive impairments in patients with persistent symptoms attributed to Lyme disease	The relationship between early-onset Lyme disease and cognitive impairment	Case-control study	n=280	Only a small number of patients with persistent symptoms linked to borreliosis show objective cognitive impairment
Andreassen, S. et al., 2023	Assessment of cognitive function, structural brain changes and fatigue 6 months after treatment of neuroborreliosis	The relationship between early-onset Lyme disease and cognitive impairment	Case-control study	n=68	Neuroborreliosis did not affect cognitive functions
Andreassen, S. et al., 2021	Cognitive function, fatigue and Fazekas score in patients with acute neuroborreliosis	The relationship between early-onset Lyme disease and cognitive impairment	Case-control study	n=72	Neuroborreliosis did not appear to affect cognitive functions in the acute stage of the disease
Haahr, R. et al., 2020	Risk of Neurological Disorders in Patients With European Lyme Neuroborreliosis: A Nationwide, Population-Based Cohort Study	Lyme disease in relation to the development of dementia	Cohort study	n=2067	Lyme neuroborreliosis patients did not exhibit increased long-term risks of dementia, Alzheimer’s disease, or Parkinson’s disease
Bransfield, R.C. et al., 2024	Late-stage borreliosis and substance abuse	Psychiatric symptoms in patients with Lyme disease	Case report	n=1	Homicide and suicide committed
Redolfi, A. et al., 2024	Retrograde and semantic amnesia in a case of post-treatment Lyme disease syndrome: did something lead to a psychogenic memory loss? A single-case study	Lyme disease in relation to the development of dementia	Case report	n=1	Impaired intensive attention, retrograde, and semantic memory. Identity loss, specific phobias, dissociative symptoms, and depressed mood
Senejani, A. G. et al., 2022	Borrelia burgdorferi Co-Localizing with Amyloid Markers in Alzheimer’s Disease Brain Tissues	Lyme disease in relation to the development of dementia	Case-control study	n=2	*B. burgdorferi* found in two out of ten patients, both in Alzheimer’s Disease brain tissues, co-localized with amyloid markers
Gadila, S.K.G. et al., 2021	Detecting Borrelia Spirochetes: A Case Study With Validation Among Autopsy Specimens	Lyme disease in relation to the development of dementia	Case report	n=1	*Borrelia burgdorferi* linked as a potential factor in Lewy body dementia
Sanchini, C. et al., 2023	A Case of Reversible Dementia? Dementia vs Delirium in Lyme Disease	Lyme disease in relation to the development of dementia	Case report	n=1	Pseudodementia observed; highlights the importance of diagnosing and treating reversible causes of dementia
Cunha, F. et al., 2022	Acute Lyme neuroborreliosis with transient aphasia – Case report and review of current knowledge	Rare neuropsychiatric presentations of Lyme disease	Case report	n=1	Acute Lyme neuroborreliosis presented with transient aphasia as a neuropsychiatric symptom
Gimsing, L.N. et al., 2020	Normal pressure hydrocephalus secondary to Lyme disease, a case report and review of seven reported cases	Rare neuropsychiatric presentations of Lyme disease	Case report	n=1	Secondary Normal Pressure Hydrocephalus associated with chronic infection with *Borrelia burgdorferi*, with symptoms resolving after antibiotic treatment.
Vieira, J.P. et al., 2019	*Borrelia lusitaniae* Infection Mimicking Headache, Neurologic Deficits, and Cerebrospinal Fluid Lymphocytosis	Rare neuropsychiatric presentations of Lyme disease	Case report	n=1	Word-finding difficulties and paraphasic speech observed, with preserved language comprehension

## Results

### The relationship between early-onset Lyme disease and cognitive impairment

Neuroborreliosis does not seem to affect the cognitive functions [15, 16], nor does it change the cortical thickness or brain volume in the acute stage of the disease, and, after adequate treatment, neuroborreliosis prognosis regarding the cognitive function is favorable [16]. People suffering from LD can show cognitive impairments such as attention and memory deficits [17]. However, only a small percentage of patients (2.9%) with borreliosis-attributed persistent symptoms have objective cognitive impairment [18]. The most frequently reported subjective cognitive symptom disturbances were persistent attention problems (84%), brain fog (84%), unfocused concentration (81%), working (78%) and recent (77%) memory problems, difficulty prioritizing multiple tasks (76%), word finding difficulties (76%), impaired multitasking (74%) [19]. In comparison, in a 2022 study by Maxwell S.P. et al., 80–90% had brain fog and difficulty concentrating, 70–80% had short-term memory impairment, difficulty thinking/concentrating/reading, and 55% had difficulty with speech [20]. In a 2020 study, Voitey M. and co-authors looked at the prevalence of functional symptoms. They found that patients with confirmed *Lyme borreliosis* (LB) reported fewer functional symptoms than those without. They were less likely to report memory impairment (4% vs. 16%) than healthy subjects [21].

### Cognitive impairment in post-treatment Lyme disease syndrome (PTLDS) patients

A study published in 2022 by Gorlyn M. and co-authors, which carried out a secondary analysis of previously reported data, looked at whether differences in speech fluency scores between groups (healthy individuals, major depressive disorder, and PTLDS patients) persisted after adjusting for the general verbal ability, processing speed, and memory performance. People with post-treatment symptoms of Lyme disease *did* perform worse than healthy volunteers. PTLDS patients showed persistent language fluency deficits even after controlling for verbal ability, processing speed, and memory difficulties, concluding that difficulties with language fluency appear to be a separate cognitive problem within the range of PTLDS symptoms [22].

In a cohort study published in 2022 by Geebelen L. et al., the subjects were divided into three groups: those with *erythema migrans*, those with disseminated/late Lyme disease, and a control group. PTLDS were present in both LB groups but with a higher percentage in the disseminated and late LB patients (20.9% vs. 5.9%). Symptoms attributed to PTLDS, such as memory problems, difficulty concentrating, and difficulty finding the right words, were not statistically significantly different between the control group and one or other form of Lyme disease groups either before or after the 6- or 12-month follow-up [23]. There are conflicting results regarding memory, with PTLDS patients responding more slowly but as accurately as controls on a working memory task. PTLDS patients also had lower functional MRI activity in the expected areas [24]. Another study assessing cognitive function 12 months after treatment found no changes in attention or processing speed [25].

### Lyme disease in relation to the development of dementia

In a study by Alireza G. Senejani and co-authors on the influence of *Borrelia burgdorferi* infection on neurodegenerative diseases, brain preparations from 6 Alzheimer’s and 4 Parkinson’s disease cadavers were investigated. The results of *Polymerase Chain Reaction* (PCR) and histochemical and immunochemical staining strongly suggested the presence of Lyme disease-causing *B. burgdorferi* aggregates co-localized with amyloid and phospho-tau markers in the hippocampal sections of two out of ten patients showing potential link between Lyme disease and chronic neurodegenerative diseases [26], such as Lewy body dementia [27]. However, a cohort study in Denmark, which included a large 30-year sample, found that Lyme neuroborreliosis patients did not have increased long-term risks of dementia, Alzheimer’s disease, or Parkinson’s disease [28].

It is important not to rush to a diagnosis without ruling out other potential diagnoses. A study by Sanchini C. and colleagues details a clinical case involving a 75-year-old man with a background of mild memory issues. He was hospitalized due to hallucinations, confusion, and aggressive behavior that did not respond to antipsychotic medications. Comprehensive testing for infectious diseases revealed a positive *Borrelia burgdorferi* serology, which the Western blot confirmed. The brilliant clinical improvement following targeted antimicrobial treatment strongly supports a diagnosis of neuroborreliosis [29].

### Psychiatric symptoms in patients with Lyme disease

It appears that people suffering from Lyme disease have significantly lower quality of life and sleep and tend to have depressive symptoms [17]. In 2021, Vargas S.E. et al. studied a convenience sample of adults with persistent post-Lyme treatment symptoms who self-reported mild symptoms of depression, anxiety, and sleep disturbances [30]. Depression [31] and anxiety [31, 32] as symptoms of Lyme disease are also mentioned in other authors’ articles.

A study published in 2020 by Bransfield R.C. et al. compared the prevalence of symptoms experienced by 100 patients before and after Lyme disease diagnosis. Patients had only minor symptoms before the infection but very often developed a wide range of acquired multisystem symptoms after infection. The most prevalent psychiatric symptoms were depression (79%), non-restorative sleep (76%), sudden mood swings (74%), hypersomnia (73%), and insomnia (72%) [19]. In comparison, in a 2022 study by Maxwell S.P. et al., about 90% of respondents had subjective anxiety and depression, 70–80% experienced panic/anxiety attacks, *Obsessive-Compulsive Disorder* (OCD), and about 60% experienced confusion and feelings of mania, hallucinations, delusions [20].

In 2021, a Danish cohort study showed that people diagnosed with Lyme disease in the hospital had an increased risk of mental disorders, affective disorders, suicide attempts, and suicide. The highest rate of mental disorders occurred within 6 months after diagnosis, and suicide rates peaked in the first 3 years. Multiple episodes of Lyme borreliosis increased the risk of cognitive and affective disorders, as well as suicide attempts, but not suicide deaths [33].

Chronic Lyme disease is linked to a range of symptoms that can increase the risk of psychoactive substance abuse and addictive diseases. A patient with multiple tick bites and increasing multisystemic symptoms experimented with the *N-methyl-d-aspartate* (NMDA) receptor antagonist phencyclidine, which counteracts the NMDA agonism caused by Borrelia infection. During *phencyclidine* (PCP) withdrawal, he committed one murder, two assaults, and suicide. It is hypothesized that PCP withdrawal may exacerbate the symptoms caused by Borrelia-induced biochemical imbalances in the brain. This combination may have significantly increased his risk of acute homicide and suicide [32].

Few studies can be found on the neuropsychiatric manifestations of Lyme disease in the pediatric population; one study published in 2019 by Rožič M. et al. in Slovenia investigated the prevalence of various symptoms in children younger than 15 years with presentation suggestive of Lyme neuroborreliosis (LNB) or confirmed Lyme borreliosis from the *Cerebrospinal Fluid* (CSF). From the disease onset up to admission, one out of 71 patients experienced behavioral changes in the *B. Garinii* group and 2 out of 42 in the *Borrelia Afzelii* group. One patient with established *B. Afzelii* experienced hallucinations [34].

On the contrary, a nationwide, population-based study in Denmark examined whether pediatric neuroborreliosis is associated with psychiatric neurodevelopmental disorders or long-term neurodevelopmental effects. Including 1,132 children with neuroborreliosis and 11,320 matched controls, the study assessed psychiatric contacts, ADHD diagnoses, learning or intellectual disabilities, and psychostimulant use. No significant associations were found, even in sensitivity analyses [35]. Additionally, a 2021 study by Tetens M.M. et al. found no long-term increased risk of psychiatric illness requiring hospitalization or prescription drugs in Lyme neuroborreliosis patients. The rise in psychiatric drug use during the first year after diagnosis suggests that most symptoms resolve quickly [36]. Furthermore, patients diagnosed with LB had fewer and shorter functional symptoms than patients without LB. They were less likely to report irritability (2% vs. 23%) and sadness (0% vs. 16%) compared to patients without confirmed LB [21].

### Rare neuropsychiatric presentations of Lyme disease

The clinical case demonstrates the link between Lyme disease and the development of severe psychiatric disorders. A 41-year-old man whose symptoms were consistent with PTLDS developed a loss of intense attention but retained selective attention. Although executive functions and short-term and anterograde memory were intact, he had significant memory loss for past events and general knowledge. The patient also experienced the loss of identity, specific phobias, dissociative symptoms, and depressed mood, highlighting the loss of identity and phobias as the symptoms most indicative of a psychogenic mechanism of amnesia [37].

The second case report describes a patient with acute Lyme neuroborreliosis presenting with transient aphasia. In short, a 44-year-old woman with acute neuroborreliosis presented with transient aphasia, fluctuations in consciousness, fever, headache, and photophobia. Neurological examination revealed involuntary orofacial movements and global aphasia but no motor deficit. The diagnosis was confirmed by a positive IgM Western-Blot test in cerebrospinal fluids, and a 21-day course of intravenous ceftriaxone was prescribed. Remarkably, the patient made an excellent neurological recovery at the start of the hospitalization and has remained symptom-free since then during follow-up visits, with no signs of post-treatment Lyme disease syndrome [38]. A similar clinical case is described in which a 15-year-old with Lyme disease exhibited word-finding difficulties and paraphasic speech but preserved language comprehension. CSF PCR was positive for *Borrelia burgdorferi sensu lato*, identifying the *B. lusitaniae* genotype. Initially treated with intravenous Ceftriaxone and Acyclovir for suspected infectious encephalitis, he completed 21 days of Ceftriaxone. He recovered completely within 24 hours, remaining asymptomatic with a normal neurological examination [39].

The fourth clinical case describes normal pressure hydrocephalus secondary to Lyme disease. A 67-year-old healthy male with a slow onset of progressive balance problems also presented unspecified dizziness, urge feeling, neck soreness, and problems concentrating and finding words with increased irritability. Initial CSF analysis was negative for oligoclonal bands but positive for Bb IgG. A repeat lumbar puncture showed increased protein, pleocytosis, positive oligoclonal bands, and IgG, leading to a diagnosis of chronic neuroborreliosis. Antibiotic treatment was initiated, and after a 1-year follow-up, the patient was symptom-free [40].

## Discussion

The findings of this review indicate that neuroborreliosis has a generally favorable prognosis, with minimal acute effects on the cognitive function or the brain structure, when treated promptly. While many patients report subjective cognitive symptoms such as memory and attention difficulties, objective impairments are rare and typically mild. Studies report a high prevalence of psychiatric symptoms among Lyme disease patients, including depression, anxiety, and sleep disturbances. However, some studies show no significant long-term increase in the risk of psychiatric illness among patients with Lyme neuroborreliosis.

Some studies suggest a potential link between *Borrelia burgdorferi* and neurodegenerative diseases. The evidence remains inconclusive, emphasizing the importance of distinguishing Lyme neuroborreliosis from other conditions. It is known that *Borrelia burgdorferi* chronic infection can co-localize with tau tangles and amyloid deposits [41] and activate complement, affect vascular permeability, and generate nitric oxide and free radicals. All these processes are involved in the pathogenesis of Alzheimer’s disease [42]. Misdiagnosis of dementia-like syndromes can lead to delayed treatment, as illustrated by cases where patients with apparent dementia showed significant improvement after antimicrobial therapy for neuroborreliosis [29]. When diagnosing a secondary dementia-like syndrome linked to Lyme neuroborreliosis, focus should be directed to general somatic symptoms, such as fever, which are typically absent in primary dementia. A CSF examination to detect intrathecal antibody production can also be helpful, especially if the patient has visited a Lyme disease-endemic area [43]. Awareness of this form of Lyme neuroborreliosis is crucial, as early antibiotic treatment can prevent lasting complications that may arise if the disease goes untreated [44].

Chronic Lyme disease has been consistently linked to an increased risk of psychiatric symptoms, particularly in the early years following the diagnosis. Conditions such as depression, anxiety, insomnia, and even more severe issues like suicide attempts and violent behavior are prevalent among these patients. While some individuals may experience long-term mental health challenges, others show significant improvement over time, thereby suggesting that variability in outcomes may be influenced by factors such as immune responses, psychological aspects, and access to treatment. Notably, one research has highlighted that the immune response to *Borrelia burgdorferi*, the causative bacterium of Lyme disease, is not uniform, and this variation may explain why some patients experience recurrent or prolonged symptoms while others do not [45]. Genetic studies further corroborate this relationship, with immune-regulatory genes being identified as significant contributors to the development of severe mental illnesses in Lyme patients [46, 47]. Chronic pain associated with Lyme arthritis or persistent post-treatment Lyme disease symptoms often exacerbates psychological distress, contributing to heightened anxiety, depression, and other mental health issues [48]. Additionally, studies show that hospitalization due to infections increases the likelihood of a mental disorder diagnosis by 84% and the use of psychotropic medications by 42% [49]. It is also known that a prior autoimmune disease increased the risk of schizophrenia by 29% and any history of hospitalization with infection increased the risk of schizophrenia by 60%. When the two risk factors were combined, the risk of schizophrenia was increased even further [50]. These findings underscore the importance of early intervention in managing the psychiatric risks associated with Lyme disease, emphasizing the need for a comprehensive, multidisciplinary approach to care.

Lyme disease is often described as the ‘great imitator’, and the symptoms are not specific and have aetiological and phenomenological overlap with myalgic encephalitis/chronic fatigue syndrome [51] or just chronic fatigue syndrome, fibromyalgia and other chronic pain syndromes [52]. An example of how Lyme disease can be confused with multiple sclerosis is that both can have similar symptoms, such as cognitive impairment, fatigue, or paresthesias [53]. Also, in Lyme disease, white matter hyperintensities may be observed on *Magnetic Resonance Imaging* (MRI), although the lesions do not usually have the same distribution as those seen in multiple sclerosis [54]. However, no specific laboratory tests are available for the diagnosis of multiple sclerosis [55], whereas specific antibodies are used to diagnose Lyme disease [56]. Although imaging and cerebrospinal fluid tests can help differentiate between the two diseases, this underlines the challenges of differential diagnosis. Lyme disease syndrome is also known to have features very similar to post-infectious fibromyalgia after treatment [57]. Still, Lyme disease is more often characterized by migratory pain associated with tick exposure [58]. It is important that patients presenting for screening for Lyme disease due to symptoms of chronic generalized musculoskeletal pain following a tick bite should also be screened for fibromyalgia so that they can be treated appropriately [57]. Although symptoms of severe fatigue and cognitive difficulties have been observed in both chronic fatigue syndrome and post-Lyme syndrome patients, post-treatment Lyme patients have more pronounced cognitive impairment than patients with chronic fatigue syndrome compared to a healthy control group. This is particularly evident among patients with post-treatment Lyme syndrome who do not have a psychiatric illness [59].

In terms of treatment for *Post-Treatment Lyme Disease Syndrome* (PTLDS), one systematic review found no statistically significant difference between antibiotics and placebo in improving the quality of life, cognition, and depression, with antibiotics actually leading to more adverse events. Consequently, it is recommended that patients suspected of having PTLDS should not be treated with antibiotics, as the condition lacks effective disease-targeting therapies, relying instead on symptom management [60]. Furthermore, recent studies suggest that PTLDS may be a result of an autoimmune condition triggered after antibiotic treatment [61], and the overdiagnosis rates for PTLDS are alarmingly high, ranging from 80% to 100%, with new diagnoses often encompassing psychiatric, rheumatological, or neurological conditions. Long-term anti-infective treatments also lead to adverse events, including emergency department visits and hospital admissions [62]. While antibiotics like Ceftriaxone have proven effective in aiding recovery and emphasizing the importance of early diagnosis to reduce long-term mental and neurological effects, evidence remains limited on whether *B. burgdorferi* persists and continues to cause symptoms or triggers a dysregulated immune response [63]. Moreover, although some studies indicate that Lyme disease may lead to neuropsychiatric symptoms, there is a consensus in the literature that these symptoms are often overestimated and may be caused by psychological stress or co-morbidities unrelated to *B. burgdorferi* infection [64], which are sometimes overtreated [65]. There is ongoing debate on the efficacy of additional treatments such as antibiotics or intravenous immunoglobulin for patients with persistent Lyme disease symptoms, with some suggesting no proof of benefit [63], while others argue that Ceftriaxone may be effective for treating PTLDS [66].

Our study has several limitations. First, we conducted our search exclusively in the *PubMed* database by using a specific set of keywords, which may have limited our ability to identify all relevant studies. Additionally, our search was restricted to publications from 2019 to 2024, although we referenced some older studies in the discussion that were relevant to the context. While this approach ensured the inclusion of the most recent studies in the results, reflecting current diagnostic practices, it may have also excluded older research that could provide further insights into the neuropsychiatric aspects of Lyme disease. It is also important to note that eight of the studies included in our results were based on only one or two cases, accounting for nearly one-third of our findings. These studies primarily focused on rare neuropsychiatric disorders and associations with dementia. Therefore, our summarization in these areas should be interpreted with even greater caution.

## Conclusion

Our findings suggest that the relationship between Lyme disease and neuropsychiatric symptoms remains uncertain, with research yielding mixed results. Studies on psychiatric symptoms are inconsistent, with some indicating a possible link between Lyme disease and an increased risk of psychiatric disorders, such as depression or anxiety, while others, including those focusing on children, find no significant association. This variability underscores the need for further research to clarify these potential connections. Regarding cognitive function, Lyme disease does not appear to cause significant impairments, though some patients, particularly those with post-treatment Lyme disease syndrome, report cognitive dysfunctions. However, objective cognitive deficits seem to be rare, and large-scale studies have not found any increased risk of dementia. While no definitive link between Lyme disease and neurodegenerative disorders has been established, isolated cases resembling dementia-like syndromes highlight the importance of thorough differential diagnosis. While significant progress has been made in understanding the neurological and psychiatric impacts of Lyme disease, gaps remain in diagnostic precision, treatment efficacy, and the long-term understanding of its pathophysiology. Studies emphasize the need for collaborative, multidisciplinary strategies to enhance patient care and address the complex neuropsychiatric effects of the disease. Continued research into the pathophysiology, differential diagnosis, and optimal treatment strategies is critical to improving outcomes for patients affected by this multifaceted illness.

## Data Availability

Not applicable.
